# Postoperative Radiotherapy after Radical Prostatectomy: Indications and Open Questions

**DOI:** 10.1155/2012/963417

**Published:** 2012-02-28

**Authors:** Pirus Ghadjar, Daniel Zwahlen, Daniel M. Aebersold, F. Zimmermann

**Affiliations:** ^1^Department of Radiation Oncology, Inselspital, Bern University Hospital, University of Bern, Freiburgstrasse, 3010 Bern, Switzerland; ^2^Department of Radiation Oncology, Kantonsspital Graubünden, 7000 Chur, Switzerland; ^3^Department of Radiation Oncology, University Hospital Basel, 4031 Basel, Switzerland

## Abstract

Biochemical relapse after radical prostatectomy occurs in approximately 15–40% of patients within 5 years. Postoperative radiotherapy is the only curative treatment for these patients. After radical prostatectomy, two different strategies can be offered, adjuvant or salvage radiotherapy. Adjuvant radiotherapy is defined as treatment given directly after surgery in the presence of risk factors (R1 resection, pT3) before biochemical relapse occurs. It consists of 60–64 Gy and was shown to increase biochemical relapse-free survival in three randomized controlled trials and to increase overall survival after a median followup of 12.7 years in one of these trials. Salvage radiotherapy, on the other hand, is given upon biochemical relapse and is the preferred option, by many centers as it does not include patients who might be cured by surgery alone. As described in only retrospective studies the dose for salvage radiotherapy ranges from 64 to 72 Gy and is usually dependent on the absence or presence of macroscopic recurrence. Randomized trials are currently investigating the role of adjuvant and salvage radiotherapy. Patients with biochemical relapse after prostatectomy should at the earliest sign of relapse be referred to salvage radiotherapy and should preferably be treated within a clinical trial.

## 1. Introduction

Radical prostatectomy (RP) provides excellent cancer control in patients with localized prostate cancer. However, half of all patients present with one or more risk factors for recurrent disease including higher Gleason Score, extracapsular extension (TNM tumor classification pT3a), invasion of the seminal vesicles (pT3b), or positive resection margins (R1). As a result, the risk of biochemical relapse is approximately 15–40% 5 years after RP [1, 2] and still increasing later [3] with even higher significance for patients with initially markedly elevated prostate-specific antigen (PSA) values [4, 5]. In patients with biochemical relapse, median time to bone metastasis is 8 years [6]. It is more pronounced with PSA doubling time of <12 months, resulting in a 5-year metastatic progression-free survival of less than 20% [7]. From several trials, nomograms have been created to assess the risk of an individual patient for tumor progression [8, 9]. This documents the importance of adequate selection of men after curative intended local treatment of prostate cancer.

Postoperative radiotherapy (RT) can be performed directly after RP based on risk factors (adjuvant RT), or it is performed in case of biochemical relapse after RP or in patients who have persistently detectable PSA levels postoperatively (salvage RT). Three randomized controlled trials investigating the role of adjuvant RT demonstrated improved biochemical control rates [10–12], whereas metastasis-free survival and overall survival were improved in only one trial after 12.7 years of followup [13]. In contrast, to date, improved biochemical control for salvage RT has been shown only in retrospective studies.

Despite the lower level of evidence, salvage RT in patients with biochemical recurrence as compared to adjuvant RT in all high-risk patients may avoid side effects in at least a subgroup of patients being already cured by surgery alone and is therefore the preferred postoperative treatment option in many centers. Five years after RP approximately 45–54% of patients with risk factors remain without evidence of disease without adjuvant RT. This can be estimated from the control arms of the three randomized adjuvant RT trials [10–12]. This paper will provide recommendations regarding the management of prostate cancer patients, who either have risk factors for recurrence or already established biochemical relapse after RP.

## 2. Materials and Methods

Data for this paper were identified by searches of MEDLINE, Current Contents, PubMed, and references from relevant articles using medical subject headings including prostate cancer, postoperative, radiotherapy, adjuvant, and salvage.

## 3. Results and Discussion

### 3.1. Adjuvant Radiotherapy

Three randomized phase III trials have addressed the benefit of adjuvant RT to the prostatic bed in an immediate postoperative period [10–12]. The studies are summarized in Tables 1 and 2 as well as below. Two trials have been initiated in the late eighties and early nineties of the last century, using old 2 D radiation techniques, and none of these trials provided details of the performed lymph node dissection. Additionally, two out of three trials allowed the inclusion of patients with postoperatively elevated PSA values beyond 0.2 ng/mL, which nowadays is defined as PSA recurrence and therefore represents early salvage RT as compared to adjuvant RT. Therefore, data and discussion of these trials have to be interpreted carefully, and can only be transferred into modern radiation oncology with caution.

### 3.2. EORTC 22911 Trial

The European Organisation for the Research and Treatment of Cancer (EORTC) trial 22911 [10] included 1005 patients between 1992 and 2001 either having had pT3a, pT3b, or R1. Patients were randomized to undergo either RT to the prostatic fossa to a dose of 60 Gy at a median time of 90 days after surgery or observation, respectively. The primary endpoint was biochemical progression-free survival (bPFS). Biochemical relapse was defined as a PSA ≥ 0.2 ng/mL above postoperative nadir. Initial results were published after a median followup of 5 years in 2005. Postoperative RT demonstrated a significant improvement in bPFS compared to observation (74% versus 52.6%, hazard ratio (HR) 0.48, 98% confidence interval (CI) 0.37–0.62; *P* < 0.0001). There was also a significant reduction in the cumulative incidence of locoregional recurrence at 5 years of 5.4% in the RT arm and 15.4% in the observation arm, respectively (*P* < 0.0001). 

In conclusion, this trial provided strong evidence that adjuvant RT improved bPFS and local tumour control. Subgroup analysis after a central pathology review demonstrated that patients with positive resection margins benefitted most from adjuvant RT [14]. But this conclusion was only achieved by a detailed central pathological review, emphasizing the disagreement both on the detection of positive margins and definition of differentiation of the tumor by different pathologists. Treatment was well tolerated. There was no late grade 4 toxicity reported and the 5-year actuarial incidence of late grade 3 toxicity was 4.2% in the RT group and 2.6% in the observation arm (*P* = 0.07).

### 3.3. SWOG 8794 Trial

The Southwest Oncology Group (SWOG) 8794 trial [11] included 425 pT3a, pT3b or R1 patients between 1988 and 1997. Patients received either adjuvant RT to the prostatic fossa with 60–64 Gy or were observed. Postoperative PSA values were available for 376 patients (88%), and 33.8% of patients had PSA values of 0.2 ng/mL and above. Primary trial endpoint was metastasis-free survival. After a median followup of 12.7 years [13], adjuvant RT improved the median metastasis-free survival for 1.8 years (HR 0.71, 95% CI 0.54–0.94; *P* = 0.016) as well as the median overall survival for 1.9 years (HR 0.72, 95% CI 0.55–0.96; *P* = 0.023), respectively. There was no subgroup in the subset analysis on seminal vesicle infiltration, Gleason Score, or postoperative PSA value without an improvement by adjuvant RT. However, patients with a postoperative PSA > 0.2 ng/mL had a significantly higher risk for metastasis and death (*P* = 0.03), although the relative improvement of immediate postoperative irradiation was still the same. This stresses the value of early adjuvant RT before development of PSA recurrence. In contrast, it may also support the idea that there are risk factors for tumor progression which cannot be influenced by RT. Unfortunately clinical outcome of the subgroup of patients in the observational arm who ultimately received salvage RT for either increasing PSA or a local recurrence (70 patients, mean PSA value 1.0 ng/mL) is not provided. This would allow to further characterize the effect of delayed postoperative RT. Adjuvant RT was well tolerated; however, urinary incontinence was more common after RT (6.5%) compared to observation (2.8%) (*P* = 0.11), and rectal complications including proctitis and rectal bleeding were only present in the RT arm (3.3% versus 0%, *P* = 0.02). In the initial analysis, the incidence of urethral strictures was significantly higher in the RT arm (17.8%) as compared to the observation arm (9.5%) (*P* = 0.02) [11]. In contrast, adjuvant RT did not negatively impact erectile dysfunction [15].

### 3.4. ARO 96-02/AUO AP 09/05 Trial

The “Arbeitsgemeinschaft Radiologische Onkologie (ARO) und Urologische Onkologie (AUO)” of the German Cancer Society included 388 pT3-4pN0M0 patients into the trial [12]. As an unique inclusion criteria, all 266 eligible and evaluable patients had undetectable PSA (<0.1 ng/mL) postoperatively. RT of the prostatic fossa with 60 Gy was compared to observation. Primary trial endpoint was progression-free survival (PFS) defined as two consecutive PSA raises above the detection limit of the test used, local or distant recurrence, or death. The PFS rates, based on a median followup of 53.7 months, were significantly improved in the adjuvant RT arm compared to observation (72% versus 54%, HR 0.53, 95% CI 0.37–0.79; *P* = 0.0015). In an unplanned subgroup analysis, positive resection margins, absence of seminal vesicle invasion, and a preoperative PSA > 10 ng/mL defined populations with a nominally significantly proven efficacy of RT, irrespective of the tumors differentiation. The toxicity rates were low. No patient experienced grade 4 toxicity. Two patients in the RT arm developed a urethral stricture.

### 3.5. Salvage Radiotherapy

Whereas an increase of PSA after RP of a nonorgan confined cancer can be seen in approximately 15–40%, a pure local recurrence is predominant with a slow slope of PSA (>1 year after resection; PSA doubling time >12 months; PSA increase within 12 months <0.75 ng/mL), a better differentiated cancer (Gleason Score < 8), positive margins, and negative pelvic lymph nodes [9, 16]. Outside of clinical trials, a precipitated start of hormonal therapy can be avoided at least for patients carrying all favourable risk factors (PSA < 2.0 ng/mL, Gleason Score 4–7, positive resection margins, PSA doubling time >12 months) [9, 17]. Thereby, the distinction of local versus systemic tumor progression is not compromised, and additional side effects can be avoided. So far, the combination of postoperative RT and hormonal therapy has not been shown to improve overall survival as compared to postoperative RT alone [9, 17]. Taking this into account, the authors are sceptic on the value of the recent EORTC trial (NCT00949962) on adjuvant RT in stage I–III prostate cancer, randomizing 6 months of hormonal therapy in addition to adjuvant RT to analyze its impact on bPFS. If hormonal therapy is involved, overall survival seems to be a more appropriate endpoint. In principle, by irradiation, the PSA can by decreased to nonmeasurable values in up to 50% of the cases at 5-year followup [9, 18]. The observation from large retrospective trials as well as from the randomized trials of SWOG and EORTC suggests the need to start salvage RT at the earliest sign of biochemical failure, with PSA value being between 0.2 and 0.5 ng/mL [18–20]. The development of a measurable local recurrence should be avoided, because in these patients outcome after salvage RT seems to be worse [18, 21, 22]. Unfortunately, there are only retrospective studies available addressing the benefit of salvage RT (Table 3). Trock et al. analyzed 635 patients undergoing RP in the years of 1982 to 2004 who experienced biochemical and/or local recurrence and received either no salvage treatment (*n* = 397), salvage RT alone (*n* = 160), or salvage RT combined with hormonal therapy (*n* = 78), respectively [17]. Median dose was 66.5 Gy for patients with salvage RT and 67.2 Gy for patients receiving salvage RT and hormonal therapy. After a median followup of 6 years, salvage RT alone was associated with a 3-fold increase in prostate-cancer-specific survival compared to those with no salvage treatment (HR 0.32, 95% CI 0.19–0.54; *P* < 0.001). Use of hormonal therapy was not associated with additional increase in prostate-cancer-specific survival. The increase in prostate cancer-specific survival associated with salvage RT was most marked in men with a PSA doubling time of less than 6 months and in patients with a Gleason Score of 8–10. This is an important finding as it suggests that patients with prostate cancer and adverse risk factors profit most from salvage RT. It is in contrast to other data, emphasizing the higher probability of a local tumor growth with long PSA doubling time and better differentiated cancer, and therefore a better and longer lasting effect on biochemical control [23, 24]. Therefore, until results from randomized trials become available, it seems not justifiable to refuse the admittance of salvage RT to any subgroup of patients.

A large multi-institutional retrospective study by Stephenson et al. analyzed 1540 patients who underwent salvage RT for biochemical relapse between 1987 and 2005 [9]. The primary study endpoint was defined as disease progression after salvage RT with PSA value ≥0.2 ng/mL above post-RT nadir, initiation of systemic therapy, or clinical progression. A total of 214 patients (14%) received neoadjuvant and/or concurrent hormonal therapy for a median duration of 4.1 months. Median RT dose was 64.8 Gy. The median followup was 53 months. Overall, the 6-year progression-free probability (PFP) after salvage RT was 32%. However, when analyzed according to PSA levels at initiation of treatment, an estimated 48% who received salvage RT without hormonal therapy at a PSA level ≤0.50 ng/mL were disease-free at 6 years compared to 40%, 28%, and 18% of those treated at PSA levels of 0.51–1.00, 1.01–1.50, and greater than 1.50 ng/mL, respectively. These results suggested the need for salvage RT at the earliest sign of biochemical relapse after RP. The 4-year PFP estimates after salvage RT alone were still improved in patients with high-risk features such as PSA ≥ 2 ng/mL before salvage RT, Gleason Score of 8–10, and PSA doubling time of ≤10 months [9, 24]. However, data from randomized controlled trials are lacking. 

Overall, most clinical data support that a high percentage of patients with rising PSA after RP have a local recurrence. RT to the prostatic bed alone allows long lasting tumor control, avoiding the toxicity of pelvic lymphatic irradiation and additional hormonal therapy as well. It seems reasonable to follow the guidelines on target volume definition from the EORTC Radiation Oncology Group, to reach an optimal compromise on both target volume coverage and sparing of critical organs and structures at risk. For the authors, it is a prerequisite to use such guidelines not only for participating in clinical trials on prostate cancer, but when highly sophisticated modern RT techniques with steep dose decrease close to the margins of the planning target volume are being used, in order to prevent out volume or marginal local recurrences [34].

### 3.6. Effect of Dose Escalation

In accordance with the well-described dose-escalation trials for primary RT of localized prostate cancer [35], it has recently been proposed that dose intensification either for salvage RT [29, 36] or adjuvant RT [37] would be more effective in terms of cancer control. From very recent retrospective reports, it became obvious that total doses of more than 66 Gy can be used safely when modern techniques are available [26, 27, 29, 36]. Nevertheless, intensity-modulated RT to the prostate bed-up to 75 Gy was associated with 30% late grade 2 genitourinary toxicity [27].

Also, it has been suggested that each Gy increase in total dose may improve the biochemical tumor control by more than 3%, having doses between 64 and 70 Gy still in the steep part of the dose-response curve [29]. Therefore, a total dose towards 70 Gy might be considered in salvage situation, when the risk of severe toxicity can be minimized by using modern radiation techniques. In the absence of results from randomized trials, the potentially improved local tumor control by higher RT dose should be carefully weighted out against possibly increased toxicity. In principle, toxicity of salvage RT with total doses of about 70 Gy is low with less than 3% of late grade 3 proctitis or genitourinary side effects, respectively. [19, 27, 33, 38].

However, the dose-dependent effect has never been prospectively assessed both in the adjuvant or salvage setting. The Swiss Group for Clinical Cancer Research (SAKK) is conducting a randomized controlled international trial comparing salvage RT with 64 Gy and 70 Gy without hormonal therapy in patients with prostate cancer and biochemical relapse after RP (SAKK 09/10, NCT01272050). The trial will include men ≤75 years with pT2-3 N0 R0-1, with a PSA of at least ≥0.1 ng/mL and rising but ≤2 ng/mL. Patients with evidence of macroscopic recurrence or metastatic disease are excluded. It is estimated to enroll 350 patients, and the trial is currently recruiting patients. The primary endpoint is freedom from biochemical progression including a PSA of ≥0.4 ng/mL and rising and/or clinical failure. The trial will contain quality of life analysis, quality assurance of RT, and a central pathology review.

### 3.7. Adjuvant versus Salvage Radiotherapy

The advantages of immediate adjuvant RT are obvious from three randomised trials, achieving a biochemical control at 5 years of more than 20% higher than from salvage RT [10–12, 39]. Nevertheless, it has to be taken into account that in two out of three trials on adjuvant RT, more than 25% of the included patients had a PSA of more than 0.2 ng/mL at the initiation of RT which corresponds to a “salvage-like” situation [10, 11]. Importantly, the alternative concept of salvage RT avoids treatment of patients without tumor progression after RP despite having risk factors such as R1 or pT3b. The toxicity and morbidity of urethral stenosis and incontinence can be abstained by starting RT years after full recovery from RP.

There have been multiple retrospective clinical trials comparing the influence of either immediate adjuvant or salvage RT on local control and/or biochemical control [40]. Consistent improvements in both endpoints have been observed. The biochemical control at 5 years was approximately 69–89% for adjuvant and only 39–68% for salvage RT. Local control rates were higher than 95% for adjuvant and 79–93% for salvage treatment. Of course, this can be explained, at least in part, by a selection bias in favour of adjuvant RT, since it is known that roughly half of the patients who underwent adjuvant RT would not experience a PSA recurrence even without RT; moreover, the PSA values in the salvage situation are higher, indicating a higher tumor load demanding higher radiation doses.

Three randomized clinical trials are currently comparing the timing of RT to answer if immediate adjuvant policy will be any more effective than a salvage policy. The RADICALS (radiotherapy and androgen deprivation in combination after local surgery) trial (NCT00541047) conducted by the Medical Research Council (UK) and the National Cancer Institute of Canada Clinical trials Group aims to recruit 4000 patients; primary endpoint is 10-year prostate-cancer-specific survival. The trial has two randomizations steps, one regarding the time of RT with a dose of 66 Gy to the prostatic fossa and one regarding use of combined six months hormonal therapy with postoperative RT.

The French Groupe d' Étude des Tumeurs Uro-Génitales (GETUG) has activated the GETUG-17 trial (NCT00667069) comparing adjuvant versus salvage RT combined with 6 months of hormonal therapy. The estimated enrolment is 718 patients, and the primary endpoint is event-free survival (including biochemical progression) at 5 years.

Finally, the RAVES (radiotherapy adjuvant versus early salvage) trial (NCT00860652) has been activated by the Tasman Radiation Oncology Group (TROG) comparing adjuvant versus salvage RT (64 Gy) without hormonal treatment. The estimated enrollment is 470 patients, and the primary endpoint is biochemical failure.

Until results of these trials become available, the optimal timing and dose of postoperative RT and the value of additional hormonal therapy remain unknown.

Nevertheless, based on evaluation of risk factors for biochemical or clinical failures coming from previous retrospective analysis and three recent randomized trials, most patients with R1, pT3 disease may be offered immediate postoperative RT [10, 12, 13]. Besides, patients with preoperative PSA values of more than 10 ng/mL and a preoperative PSA velocity of >2 ng/mL per year may also benefit from immediate adjuvant RT in terms of improved biochemical control, but this has not been proven by randomized trials [3, 4, 12, 14, 41]. For all the other patients carrying less pronounced risk factors for tumor recurrence, it seems more reasonable to balance the superior initial quality of life and the lower risk of clinical or PSA recurrence in favour of salvage RT. A score algorithm or nomograms may help in decision making [6, 9, 42] using risk factors for recurrence as seminal vesicle infiltration, Gleason Score, and pre-RT PSA-value.

On the other hand, the natural course and life expectancy of men besides prostate cancer have to be considered, with its enormous global inequality [43]. With a realistic life expectancy of less than 5 years, further treatment should not be offered in the adjuvant situation, and even with a life expectancy of up to 8 years, salvage RT seems to be appropriate.

Thereby, it should be remembered that it needs about 8 years from biochemical recurrence to the development of a clinically measurable progression [6].

## 4. Conclusions

The use of adjuvant RT after RP in patients with adverse risk factors has demonstrated improved biochemical control and overall survival. However, there is a relevant risk of overtreatment as patients might be included who are cured by surgery alone. We recommend to offer adjuvant RT to all patients with R1 resection and pT3 disease and to also consider preoperative PSA values of more than 10 ng/mL and a preoperative PSA velocity of >2 ng/mL per year as additional risk factors for tumor recurrence.

Alternatively, patients can be treated with salvage RT in the event of biochemical relapse, especially when they are carrying less dominant risk factors for tumor recurrence. It is recommended that such salvage RT should be performed as early as possible, preferably with PSA values below 0.5 ng/mL, and although it was shown to be most effective in patients with adverse risk factors, it should not be withhold from any definite subgroup of patients with biochemical recurrent disease. The optimal dose and timing of postoperative RT is subject of national and international phase III trials. Outside of clinical trials, 60–64 Gy should be used in the immediate postoperative setting and 64–72 Gy in the salvage setting, dependent on the absence or presence of macroscopic recurrence. Patients with biochemical relapse after RP should be treated within clinical trials to answer open questions on dose and timing as soon as possible. 

## Figures and Tables

**Table 1:** Characteristics of randomized trials on immediate adjuvant RT.

Trial	SWOG 8794	EORTC 22911	ARO 96-02
Year of initiation	1988	1992	1996
Pats.			
Randomized Eligible Percent Median age	431 425 98.6% 64.5 years	1005 968 96.3% 65 years	307 from 385 selected 266 87.3% 64 years

Inclusion criteria	cT1-2, post-RP: Extraprostatic extension and/or seminal vesicle invasion and/or positive resection margins	Extraprostatic extension and/or seminal vesicle invasion and/or positive resection margins	cT1-3, post-RP: Extraprostatic extension and/or seminal vesicle invasion
	pT3 and/or R1, c/p N0 (97% pelvic LN-dissection) cM0	pT2 R1 or pT3 R0-1, c/p N0 (99% pN0) cM0	pT3-4 R0-1 pN0 cM0
	SWOG PS 0–2 Age not reported	WHO PS 0-1 Age <76 years	WHO PS 0-1 Age <76 years

Postop. PSA	<0.2 ng/mL: 66.2 % ≥0.2 ng/mL: 33.8 %	≤0.2 ng/mL: 88.7% >0.2 ng/mL: 10.7% Unknown: 0.6%	<0.1 ng/mL: 100%

Stratification	Positive margins or capsule invasion versus invasion of seminal vesicles versus positive margins and capsule invasion; HT	Institution; capsule invasion; positive margins; invasion of the seminal vesicles	Gleason Score; resection margins; neoadjuvant HT; tumor stage

Hormonal therapy	8.5%	10.0%	11.5%
Adjuvant RT	30–32 × 2.0 Gy	30 × 2.0 Gy (in 90.8%)	30 × 2.0 Gy (in 82%)
Time from RP to RT	<18 weeks	<16 weeks	10–30 weeks

Treatment in observation arm	RT: 33.2%	RT: 22.5% HT: 9.1% Other: 1%	Not reported

Median followup	12.6 years	5 years	4.5 years

Primary endpoint	Metastasis-free survival (bone, visceral, extrapelvic lymph nodes)	Biochemical progression-free survival	Progression-free survival

Definition of bNED	PSA > 0.4 ng/mL for postop. PSA < 0.4 ng/mL	PSA > 0.2 ng/mL above lowest postop. PSA	2 increasing PSA values

bNED: biological no evidence of disease; RP: radical prostatectomy; RT: radiotherapy; HT: hormonal therapy; PS: performance status.

**Table 2:** Results of randomized trials on immediate adjuvant RT.

Trial	SWOG 8794	EORTC 22911	ARO 96-02
Overall survival	HR: 0.72 (95% CI 0.55–0.96), *P* = 0.023	HR: 1.09 (98% CI 0.67–1.79), *P* = 0.68	Not reported

bNED	HR: 0.43 (95% CI 0.31–0.58), *P* < 0.001	HR: 0.48 (98% CI 0.37–0.62), *P* < 0.0001	HR: 0.53 (95% CI 0.37–0.79), *P* = 0.0015

Metastasis-free survival	HR: 0.71 (95% CI 0.54–0.94), *P* = 0.016	Not reported	98% versus 95.1 % (n.s.)

Clinical progression-free survival	HR: 0.62 (95% CI 0.46–0.82), *P* = 0.001	HR: 0.61 (98% CI 0.43–0.87), *P* = 0.0009	Not reported

Time to initiation of hormonal therapy	HR: 0.45 (95% CI 0.29–0.68), *P* < 0.001	Not reported	Not reported

Overall toxicity	23.8% versus 11.9%, *P* = 0.002	4.2% versus 2.6% (Grade 3; *P* = 0.07)	21.9% versus 3.7%, *P* < 0.0001

Rectal toxicity	3.3% versus 0%, *P* = 0.02	Not reported	1.4% versus 0%

Urinary stricture	17.8% versus 9.5%, *P* = 0.02	Not reported	Not reported

Total urinary incontinence	6.5% versus 2.8%, *P* = 0.11	Not reported	Not reported

HR: hazard ratio; CI: confidence interval; n.s.: not significant.

**Table 3:** Characteristics and results of retrospective reports on salvage RT.

First author	Year	No. Pats.	Median pre-RT-PSA (ng/mL)	HT (%)	Med. RT-dose (Gy)	RT technique	Followup (months)	bNED (%)
Anscher et al. [25]	2000	89	1.4	9	66.0	2 D/3 D	48	50 (4 y.)
Bernard et al. [26]	2010	364	0.6	0	64.8	2 D/3 D	72	50 (5 y.)
De Meerleer et al. [27]	2008	87	0.7	56	75.0	IMRT	30	67 (5 y.)
Do et al. [28]	2002	73	2.8	9	64.8	2 D	42	45 (10 y.)
King and Spiotto [29]	2008	84	0.45	57	70.0	2 D/3 D/IMRT	>60	58 (5 y.)
Loeb et al. [30]	2008	107	~0.7	0	63.0	IMRT	53	55 (7 y.)
MacDonald et al. [22]	2004	102	1.1	0	65.8	n.r.	50	38 (5 y.)
Neuhof et al. [20]	2007	171	1.1	29	60–66	3 D	39	35 (5 y.)
Pazona et al. [31]	2005	223	0.8	4.5	63	3 D	56	40 (5 y.)
Pisansky et al. [32]	2000	166	0.9	4	64.0	2 D/3 D	52	46 (5 y.)
Stephenson et al. [24]	2004	501	0.7	17	64.8	2 D/3 D/IMRT	45	45 (4 y.)
Stephenson et al. [9]	2007	1540	1.1	14	64.8	2 D/3 D/IMRT	53	32 (6 y.)
Trock et al. [17]	2008	160	0.7	0	66.5	2 D/3 D	72	89 (10 y. OS)
Van Der Poel et al. [33]	2008	41	2.15	7	60–70	n.r.	73	44 (10 y.)
Wiegel et al. [19]	2009	162	0.33	0	66.0	3 D	41	54 (3.5 y.)

RT: radiotherapy; HT: hormonal therapy; Med. RT dose: median total dose of radiation therapy; bNED: biochemical no evidence of disease; n.r.: not reported; 2 D: 2-dimensional treatment planning; 3D: 3-dimensional treatment planning; IMRT: intensity-modulated radiation therapy; OS: overall survival.
